# Community Pharmacy Practice in Italy during the COVID-19 (SARS-CoV-2) Pandemic: Regulatory Changes and a Cross-Sectional Analysis of Seroprevalence

**DOI:** 10.3390/ijerph18052302

**Published:** 2021-02-26

**Authors:** Francesca Baratta, Giulio Mario Visentin, Lorenzo Ravetto Enri, Marco Parente, Irene Pignata, Francesco Venuti, Giovanni Di Perri, Paola Brusa

**Affiliations:** 1 Department of Drug Science and Technology, University of Turin, Via Pietro Giuria 9, 10125 Turin, Italy; giuliomario.visentin@unito.it (G.M.V.); lorenzo.ravettoenri@unito.it (L.R.E.); irene.pignata@unito.it (I.P.); paola.brusa@unito.it (P.B.); 2 Turin Pharmacy Owners Association “Federfarma”, Via Sant’Anselmo14, 10125 Turin, Italy; marcoparen@yahoo.it; 3 Department of Medical Sciences, Unit of Infectious Diseases, University of Turin, C.so Svizzera, 164, 10149 Turin, Italy; f.venuti@unito.it (F.V.); giovanni.diperri@unito.it (G.D.P.)

**Keywords:** community pharmacies, pharmacists, COVID-19, SARS-CoV-2, Italy, seroprevalence, safety measures

## Abstract

Pharmacists in the community and the essential requirement to safeguard their own health have become fundamental since the spread of the severe acute respiratory syndrome coronavirus 2 (SARS-CoV-2). The aims of this paper were (I) to analyze the directives provided to pharmacists in 2020 regarding preventative safety measures to be adopted; (II) to determine the number of pharmacists who came into contact with SARS-CoV-2 in North-West Italy and relate this to the adopted preventative measures. The first aim was pursued by conducting a bibliographic research, consulting the principal regulatory sources. The second one was achieved with an observational study by administering a questionnaire and performing a serological test. The various protection measures imposed by national and regional legislation were analyzed. Two hundred and eighty-six pharmacists (about 8% of the invited ones) responded to the survey. Ten pharmacists reported a positive result to the serological test. Of the subjects who presented a positive result, three declared that they had not used a hand sanitizer, while two stated that they had not scheduled the cleaning and decontamination of surfaces. Two interviewees had not set up a system of quota restrictions on admissions. In four cases, a certified cleaning company had decontaminated the premises. The results of our study show that during the coronavirus disease 2019 (COVID-19) pandemic, the most pressing challenge for community pharmacists has been the protection of staff and clients inside the pharmacy; the challenge to be faced in the near future will probably be the management of new responsibilities.

## 1. Introduction

Community pharmacies are among the most accessible health services available to the public and often represent the first point of contact for a patient seeking general health advice or treatment for minor conditions [1].

Pharmacists in the community and the essential requirement to safeguard their own health in order to carry on their professional duties have become fundamental since the spread of severe acute respiratory syndrome coronavirus 2 (SARS-CoV-2), causing coronavirus disease 2019 (COVID-19), became a critical health issue.

The first case of the new coronavirus was detected in the city of Wuhan in the Chinese province of Hubei in December 2019. On 30 January 2020, the WHO formally declared the coronavirus epidemic to be a public health emergency of international proportions [2] and, on 31 January 2020, the Italian cabinet of ministers declared a state of emergency [3]. Italy was one of the first countries to be struck by this new infection, even prior to 11 March 2020, when the WHO declared that the COVID-19 epidemic had escalated to a pandemic.

The consequences for Italy as a whole and for health workers in particular represented an enormous challenge that few had faced up to that time. Among the categories of health workers in the frontline in fighting the pandemic was that of pharmacists. From the start, an ever-growing number of people turned to community pharmacies for information about the new infection, which was becoming a constant subject of media reports. This phenomenon started to increase significantly when, in February 2020, access to doctors’ surgeries and clinics was substantially restricted [4]. Consequently, the only health professionals freely available to the general public were community pharmacists. Duarte Santos, President of the Pharmaceutical Group of the European Union (PGEU), in March 2020 stated: “In this unprecedent public health emergency, the dense network of pharmacies nearby the people is playing a vital role in supporting local communities. Community pharmacists are providing high-quality reliable information to the general public, avoiding unnecessary scares and relieving pressure on the rest of the health care system” [5].

It is clear, therefore, that community pharmacies have a vital role to play in the anti-epidemic response of a community or a country by implementing preventative measures and safeguarding public health [1]. For this very reason, pharmacies in many parts of the world have remained open throughout the various lockdowns caused by the spread of the novel coronavirus. The same occurred in Italy. In parallel, however, pharmacy staff must be able to work in conditions that ensure their own safety through all necessary safety precautions [1]. Duarte Santos also stated “Pharmacists are one of the first points of contact between the patients and the healthcare system and they play a key role in the identification and management of potential cases of COVID-19. As all the other frontline healthcare providers, they need to be protected against the high risk of being exposed to the virus” [5]. Since community pharmacies are, in normal times, considered the first point of contact for cold and flu symptoms, it is likely that pharmacy staff will encounter asymptomatic or symptomatic carriers of SARS-CoV-2 and, hence, they are at a high risk of being exposed to the infection [1].

Above all, given the fact that infected individuals may not show any symptoms, it has now been established that the implementation of safety procedures to protect health workers, in particular, interpersonal distancing and the correct use of personal protection equipment (PPE), is essential. By now, many safety guidelines have been developed both nationally and internationally, but in February 2020, when the number of SARS-CoV-2-infected patients in Italy started to grow exponentially, very little information about the virus or safety procedures was available, and Italian pharmacists had to respond, week by week, as more information and directives began to circulate.

As well as concerns for their own health and that of their customers that came into the community pharmacy every day, pharmacists had to respond to the rapid evolution of the coronavirus emergency in terms of changes to regulations, which took place at an unprecedented rate. In Italy, this took the form of a phase-out of paper-based prescriptions and the introduction of e-prescription This electronically based system further reduced contact between the family doctor and the patients.

In this context, the first aim of the present work was to analyze the directives provided to pharmacists between February 2020 and September 2020 regarding preventative safety measures to be adopted for both their own safety and that of individuals entering community pharmacies and para-pharmacies (shops like drugstores). In particular, this study focused on the safety guidelines provided to the above categories in the Piedmont region (North-West Italy), the region with the second-highest number of SARS-CoV-2-positive cases in Italy [6].

Furthermore, the second aim we pursued was to quantify the number of pharmacists who had come into contact with SARS-CoV-2 in the territory in exam using a rapid serological test to detect Immunoglobulin M (IgM) and Immunoglobulin G (IgG) and relate this figure to the preventative measures effectively adopted.

The usefulness of serological tests, which give the best results when carried out at least a week after the appearance of symptoms, has been proven in many different environments. In the first place, these tests can help to establish a diagnosis of SARS-CoV-2 infection in cases in which the symptomatic individuals do not have access to a molecular test to detect SARS-CoV-2 within two weeks or the molecular test is negative despite the presence of symptoms or radiological tests indicating COVID-19. The use of antibody tests is of fundamental importance in seroprevalence investigations in the area of management of public health. Seroprevalence investigations among the general population, or a specific population as in our case, can allow understanding the clinical and epidemiological characteristics of SARS-CoV-2 through a retrospective investigation, [7].

The knowledge of these characteristics will enable us to implement correct and effective containment measures for the epidemic. 

## 2. Material and Methods

### 2.1. Regulatory Research

A bibliographic research was conducted on the legislation in force indicating the obligations that community pharmacists had to comply with concerning individual protection, pharmacy staff, and users. The research was conducted by consulting the Official Gazette of the Italian Republic and the Official Bulletin of the Piedmont region.

### 2.2. Seroprevalence Investigation

**Figure 1:**
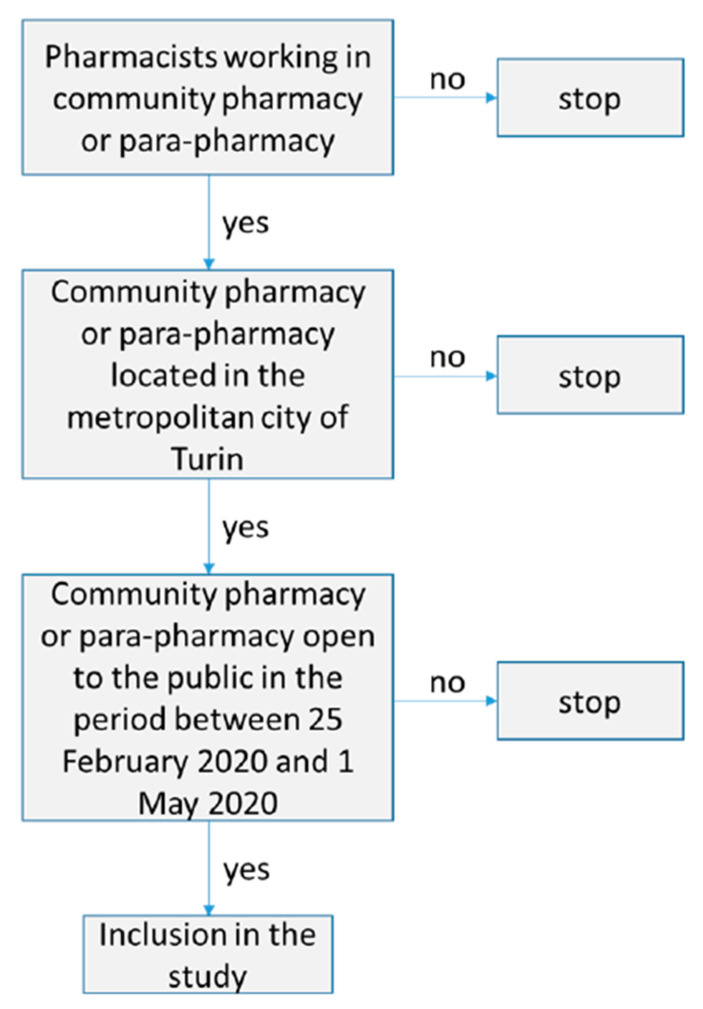
Flow chart: eligibility criteria for participants’ selection.

**Figure 2:**
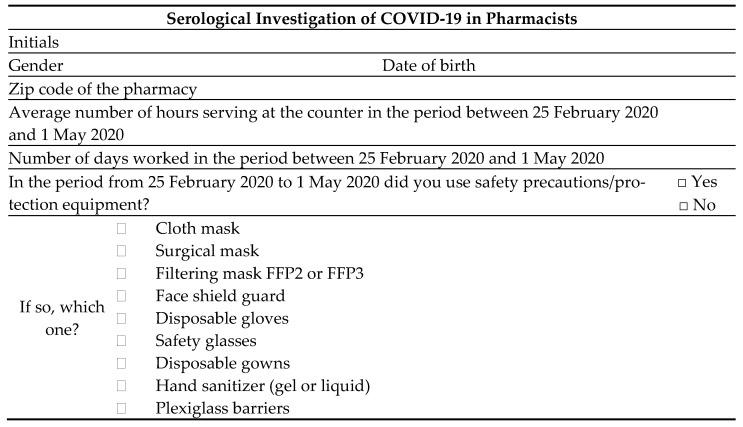
Questionnaire administered to pharmacists.

In the month of July 2020, we carried out an observational pilot study in Turin involving a serological investigation for COVID-19 which foresaw the recruitment of community pharmacists as unpaid volunteers to undergo a free serological test. Criteria for participation in the study were that volunteers had to have worked in a community pharmacy or para-pharmacy open to the public in the period between 25 February 2020 and 1 May 2020 in the province of Turin (Figure 1). The study was designed as a cross-sectional analysis of seroprevalence, representative of the population of pharmacists, mainly working in Turin (the regional capital), with a longitudinal assessment of a sub-group of that population. The pharmacists that participated in the study underwent a rapid serological test of capillary blood to qualitatively detect the presence of IgG and IgM in peripheral blood (COVID-19 IgG/IgM Rapid Test. Prima Lab. Balerna, Switzerland). That test was compared with a commercial RT-PCR test, and results demonstrated a high sensitivity and specificity (100% and 98%, respectively) [8]. If the result was positive, the subject immediately underwent a molecular test (real-time reverse-transcription polymerase chain reaction (rRT-PCR) assay) to eventually detect SARS-CoV-2. In the same circumstance, a self-report questionnaire was collected (Figure 2). The questionnaire consisted of a few, mostly closed-ended, questions about the location of the pharmacy, some demographic information regarding the pharmacists (sex and age), the number of days and hours effectively worked in contact with the public in the period in exam, as well as the safety precautions adopted and the personal protection equipment used. It was developed by a group made of medical doctors, pharmacists, university researchers, and pharmacy users. 

The sought outcomes detectable through the survey were the number of pharmacists positive to the serological test, the type of protection device used, the number of positive pharmacists using a certain protection device, the number of negative pharmacists using a certain protection device. A potential selection bias could have occurred since the recruitment of participants was carried out on a voluntary basis.

The idea was to assess the survey by doing a pilot analysis in the first wave of the pandemic in a small area and on a small sample and then to extend the survey during the next pandemic waves.

Descriptive statistics were performed. Data distribution was assessed using the Shapiro–Wilk test. The comparison between the proportions was performed by Fisher’s exact test. The level of significance was fixed at a *p*-value of 0.05 and a 95% confidence interval (CI). 

Considering the legislation in force in Italy, approval by an ethics committee was not required because the pharmacists participated in the study on a voluntary basis and they were informed of the characteristics and the purpose of the study. The questionnaire was anonymous, personal data were not collected, and there is no way to trace back the answers to a specific responder. Furthermore, even if the serological test could have been carried out directly by each involved pharmacist, to minimize a possible bias in the method of execution and to use the same model test (one of the most sensitive capillary blood tests at that time) for all involved pharmacists, it was decided to have tests performed at the same moment by the same professional worker.

## 3. Results

### 3.1. Anti-COVID-19 Preventative Measures Applied in Pharmacies in the Piedmont Region

The first case of the COVID-19 epidemic was detected in Italy on 21 February 2020 [9]. As a result, a few days later, on 24 February 2020, pharmacists in Piedmont received the first related health/hygiene recommendations. In particular, pharmacies were included among the points in which it was recommended to make hand sanitizer available for disinfecting hands to all users. Furthermore, pharmacies were recommended to inform their clients of the best practices to adopt in order to prevent the spread of the epidemic and to curb the effects of the many fake news reports in circulation: they were given the support of the specific informational material provided by the Istituto Superiore di Sanità (Higher Health Institute) and the Italian Health Ministry. This material instructed readers to wash their hands frequently, avoid close contact with people suffering from acute respiratory diseases, avoid touching the eyes, nose, and mouth, cover the mouth and nose when coughing or sneezing, avoid taking antibiotics or antiviral drugs unless prescribed by a doctor, disinfect surfaces with a chlorine- or alcohol-based disinfectant. As regards protection equipment for the respiratory tract, the instructions were to use them only when one had the suspicion of having been infected or when assisting infected people.

The next day, on 25 February, pharmacies were informed that they should remain open in accordance with their normal opening hours, making sure to prevent congestion inside the pharmacy and keeping to social distancing regulations, as already indicated by national regulations on privacy. In addition, all internships for undergraduates of Pharmacy courses were suspended with immediate effect.

On 6 March, it was advised that, in situations involving contact with others, an interpersonal distance of 1 m should be kept. Moreover, besides the health/hygiene measures mentioned above, the following instructions were provided: in geographical areas classified as high-risk, pharmacists should wear personal protection equipment (masks and gloves).

Based on the rate of infection and following a government decree of 8 March, a number of provinces in Piedmont were included in what was defined as a “red zone”, that is, a zone with a high number of infections and, therefore, a high-risk area; within this area, strict limits on movement were imposed on inhabitants, as well as other restrictions on businesses. On 10 March, the “red zone” was extended to the entire territory of Italy. Concerning the activities of pharmacies in Piedmont, the option for individual community pharmacies to continue to provide services while working behind closed doors was granted, that is to say, without allowing customers to enter the pharmacy itself. Furthermore, it was decreed that any infection of pharmacy staff members should not automatically lead to the closure of the same: in the event of a positive test result, the staff member should be quarantined, and the premises be thoroughly disinfected, but the other staff members could continue working normally, wearing masks. Points of care or telemedicine were suspended, given the impossibility of maintaining an interpersonal distance of one meter, with the exception of urgent cases or if adequate safety measures such as personal protection equipment were adopted. Finally, in light of the fact that it became compulsory to ensure a distance of at least 1 m between the pharmacist and the client, transparent plexiglass barriers were recommended as well as markings on the floor to indicate and facilitate the maintenance of a safe distance between customers. The use of masks was advised when possible, though taking into account their scarcity on the market at that time.

As of 8 April, masks and gloves became compulsory for anyone involved in sales to the public and, hence, also for pharmacists. According to the government decree of 10 April, it became obligatory that, for premises of up to 40 sqm, only one client could enter at a time, as well as a maximum of two staff members; for premises with more floor space, entry to the premises had to be regulated according to the available space and, if possible, separate entry and exit points should be instituted.

From 4 May, some restrictions on movement were relaxed within the region, and a gradual reopening of some businesses commenced. As for pharmacies, the previous measures were confirmed, with the addition that wearing masks became compulsory for the general population (with the exception of children under the age of 6 and subjects with conditions incompatible with wearing masks) in confined spaces accessible to the public; thus, in pharmacies too.

From mid-May, while maintaining the safety precautions and regulations regarding personal protection equipment already in place, it was thereafter possible to perform health services such as point-of-care service and telemedicine. Health inspections by the local health authorities, a routine check for pharmacies under the National Health Service, were suspended until 31 August 2020.

In September 2020, there was an extension, for the duration of the state of emergency, of all the measures detailed above and summarized in Table 1 [10,11,12,13,14].

### 3.2. Seroprevalence Investigation

All the approximately 3600 pharmacists enrolled in the order of pharmacists in the province of Turin were invited to participate in the survey. Two hundred and eighty-six pharmacists responded to the survey (about the 8%). The median age was 42 years (IQR = 51–32); 80% of the respondents were female. The age group that responded most frequently to the questionnaire was that of individuals between 30 and 39 years of age, accounting for 34% of the respondents, followed by the group of 40- to 49-year-old individuals, which represented 27% of the respondents; 73% of the respondents were under 49 years of age. In addition, 90% of the pharmacists that participated in the survey were employed in pharmacies located in the regional or in provincial capitals. We observed that 95% of the respondents worked in pharmacies, while 5% worked in para-pharmacies. On average, the pharmacists that took part in the survey had worked seven hours per day and 52 days in the period in exam.

Only one pharmacist declared that no personal protection measures had been adopted. The majority of pharmacists had made use of surgical masks (191) and/or filtering masks FFP2 (135); only 13 participants had used simple cloth masks. Fifty-three pharmacists declared that they had used face shields; 37 declared using safety glasses. The most common protection measure by far was the plexiglass barrier, used by 258 respondents, while 262 interviewees used to wear disposable gloves, and only one used a disposable coat; 264 participants reported using hand sanitizers. According to the survey, 260 respondents stated that surfaces in the pharmacy had been regularly sanitized; in 116 cases, the premises had been decontaminated by a certified cleaning company; finally, 268 participants responded that the pharmacy had created separate entrance and exit for the clients.

**Figure 3:**
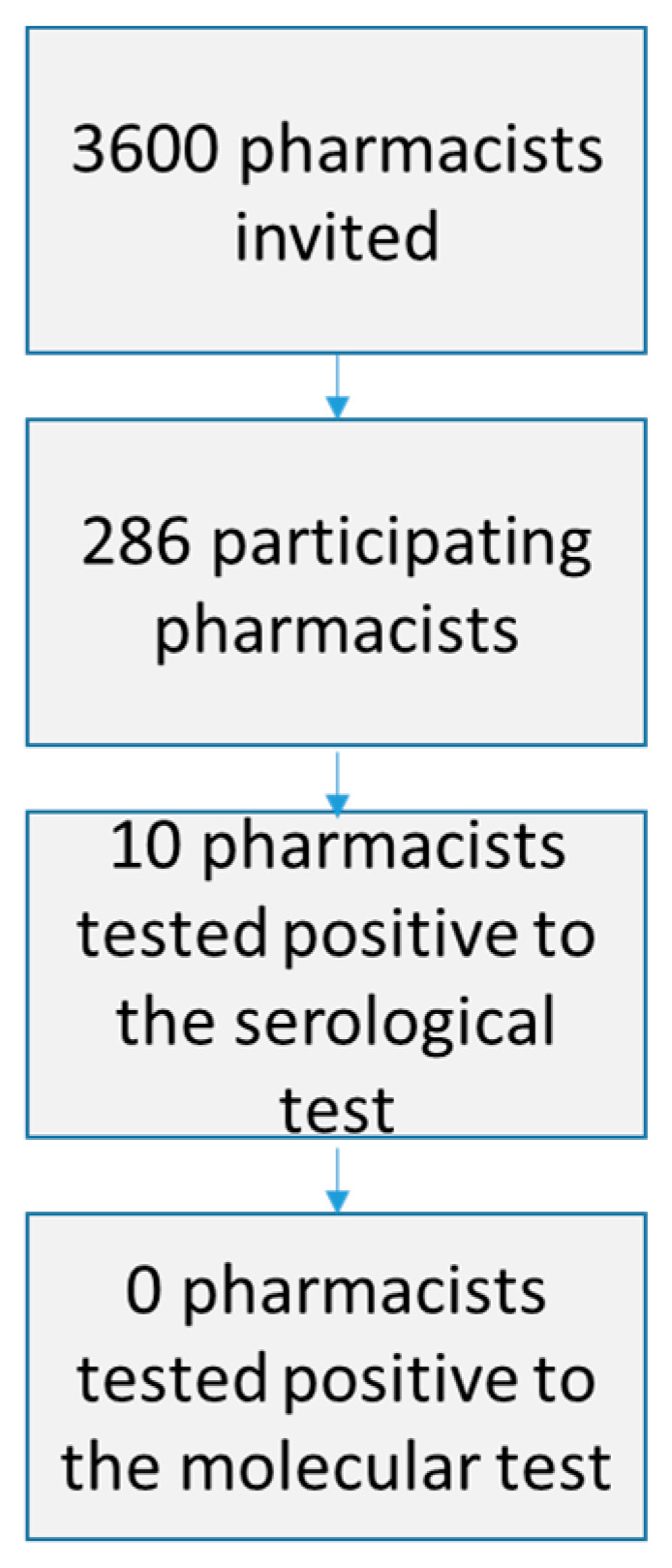
Flow chart: involvement of pharmacists and results of the serological and molecular tests.

Ten pharmacists tested positive to the serological test: no one reported a positive result to the molecular test to detect SARS-CoV-2 (rRT-PCR assay) (Figure 3). Out of the ten positive pharmacists, one was male, six were between 40 and 49 years old, one worked in a para-pharmacy, and seven had worked eight hours a day on average in the period in exam. All of these respondents had used at least one form of personal protection equipment. In particular, all of them had worn a mask of some type; nine subjects had worn gloves, and the same number had been separated from clients by a plexiglass barrier; no one had used a face shield or safety glasses. Of the subjects that reported a positive result to the serological test, three declared that they had not used a hand sanitizer, while two stated that they had not scheduled cleaning and decontamination of surfaces. Furthermore, two interviewees had not set up a system of quota restrictions on admissions. In four cases, a certified cleaning company had decontaminated the premises.

Table 2 summarizes the results obtained from the survey concerning pharmacists’ use of personal protection equipment or, more generally, the adoption of specific safety precautions. The data were subdivided into two categories: pharmacists who tested negative to the serological test and those who tested positive to the serological test. No statistically significant differences was observed between the two groups regarding the adoption of safety measures, with the exception of the use of a hand sanitizer (*p*-value < 0.05).

Table 3 presents the details of the questionnaire results for the 10 pharmacists who reported a positive result to the serological test.

## 4. Discussion

The role of the pharmacist in Italy has expanded, taking on ever-widening duties and responsibilities over the last two decades. If, in the past, the “core business” of the community pharmacy was centered on medicines, mainly the dispensation of prescribed medications, over the last few years, the focus has shifted to providing services such as the measurement of blood pressure, cholesterol, glucose, and weight, chronic disease management, early disease screening and testing, and many others. Therefore, today, the pharmacist is not only an expert in medicines, but represents an important figure in the healthcare sector who has, in collaboration with other health professionals, an important role in the provision of high-quality information that is easily understandable to the general public, as well as in notifying suspected cases of drugs’ side effects [15].

During the present coronavirus pandemic, this role has become even more essential, in that the pharmacists immediately became a point of reference capable of collaborating with the health authorities in the dissemination of accurate information to the public and in the promotion of the safe, responsible behaviors to prevent the spread of the virus [15]. Pharmacists played an exemplary role in steering the public away from inaccurate and misleading health information during the first wave of the pandemic [16], in particular, taking into account the difficulties for the general public in gaining access to their family doctor.

Moreover, community pharmacies can be a point of reference for performing tests for SARS-CoV-2 for customers (with or without the assistance of other health professionals), as well as for notifying the relevant health authorities of any detected positive cases and for supporting contact tracing.

Indeed, during the SARS-CoV-2 outbreak in autumn 2020, community pharmacies made rapid antigen detection immunoassays (RAD) widely available to the public, being these assays particularly suited for point-of care testing (POCT) and for the detection, leading to effective isolation, of COVID-19 patients. Many pharmacies set up small stands where health professionals could perform the tests in a safe and controlled environment. POCT made testing more capillary and accessible, also creating a strong collaboration between public authorities and private subjects. Even though the sensitivity and specificity of RAD are not as high as those of the RT-PCR test, these assays have proven to be accurate during the first phase of the infection [17], when SARS-CoV-2 viral load was higher [18].

In light of the above, it is most likely that the frontline position of community pharmacists will further expand with the protraction of the pandemic [1]. Considering that many SARS-CoV-2 vaccines are now available for the general population, community pharmacists are in the ideal position to establish a capillary network of vaccination points [19]. In view of this, in some parts of the world such as Australia, United Kingdom, and United States, pharmacists have already been authorized to administer SARS-CoV-2 vaccines [20,21,22]. Furthermore, the annual flu vaccination campaign performed through community pharmacies would become even more important in the context of the SARS-CoV-2 pandemic: hence, pharmacies could acquire a dual role in this situation. It is clear that vaccinating a high percentage of the population would ensure a greatly reduced incidence of the normal seasonal flu, the symptoms of which are similar to those of SARS-CoV-2 infection [23]. It should be considered that, even though research is still in its preliminary phase, the anti-influenza vaccine administered to persons over 65 years of age appears to slow the spread of COVID-19 infections among this age group and leads to a less severe clinical reaction. Decades of experience with pharmacist vaccinators all over the world has, for example, demonstrated that the ease of access to vaccination through pharmacies has significantly increased the rate of immunization in the population; patients are better informed about vaccines, and those who otherwise would never have the opportunity to be vaccinated can be reached [24,25]. Despite the relevance of these considerations, the laws in force in Italy currently do not allow pharmacists to administer vaccines.

On the other hand, should adequate safety measures not be applied, the community pharmacy could become an unwilling hub in the transmission of the new coronavirus, since it is often the first point of contact for infected patients and, hence, a high-risk area for patients with non-transmissible diseases entering the pharmacy to obtain their regular supply of medicines [1]. In addition, it is even more essential to ensure that pharmacists themselves are protected, in that the continuity of their service to the public must not be disrupted.

Published data regarding protection measures in community pharmacies amid the COVID-19 pandemic are still limited. Some studies showed that pharmacists were somewhat aware of the correct virus protection measures [26,27,28]. To our knowledge, there are still few data that can demonstrate the real efficacy of these measures in preventing infection among pharmacists. In this context, our pilot study suggests that in Italy pharmacists’ protection has been effectively regulated and respected. Furthermore, it suggests that the applied measures could be effective.

## 5. Conclusions

What has emerged from the present investigation of seroprevalence carried out in the province of Turin is that, despite the facts that the analyzed sample of pharmacists was relatively small and the answers to the questionnaire were self-reported—and notwithstanding the issues of the sensitivity of a serological test performed more than five weeks from the appearance of symptoms and the actual detection rate of cases of asymptomatic and mildly symptomatic infections—it is possible to affirm that the protective strategies put in place in pharmacies seem to have been effective in halting the spread of the virus among pharmacists. Indeed, only 10 pharmacists out of the 286 who voluntarily participated in the study reported a positive result to the serological test. It would be interesting to analyze the percentage of infections in other non-hospital health settings and in other places open to the public in order to identify eventual critical points in the management of protection and safety precautions. Furthermore, it would be necessary to carry out further studies with larger samples to confirm the results of this pilot study. Some changes can be applied to the questionnaire to better identify the most significant measures to reduce the infection.

If, so far, the most pressing challenge for community pharmacists has been the protection of staff and clients from the spread of infection inside their pharmacy [29], the challenge to be faced in the near future will probably be the management of new responsibilities and the involvement in non-emergency public health activities.

## Figures and Tables

**Table 1:** Preventative measures to be applied in community pharmacies and para-pharmacies.

**Health and hygiene measures** Wash hands frequently provide a alcohol-based detergent for hand-washing avoid close contact with persons suffering from acute respiratory infections avoid hugging and handshaking keep, in social contact, an interpersonal distance of at least 1 m practice good respiratory hygiene (sneeze or cough into a handkerchief or tissue, avoiding contact between the hands and respiratory secretions) avoid sharing water bottles and glasses, especially during sport activities avoid touching the nose, eyes, or mouth with the hands do not take antibiotics or antiviral drugs unless prescribed by a doctor clean all surfaces with alcohol-based or chlorine- based disinfectants it is strongly recommended in all social contacts to use a protection of the respiratory tract as an additional measure to the existing health/hygiene protection measures **Protection measures to protect users** Provide adequate information about protection measures Implement rules of access in order to prevent congestion and ensure an interpersonal distance of at least 1 m between customers. For premises up to 40 sqm, only one person may enter at a time, as well as two assistants; for premises larger than 40 sqm, access is regulated according to the floor space available; separate entry/exit points should be instituted Ensure that an ample supply of hand sanitizer is available and easily accessible at all times: the sanitizer should be a disinfectant; encourage all customers and staff to use the sanitizer In cases where self-service is allowed or products may be handled by customers, all customers must disinfect their hands with a hand sanitizer. Alternatively, customers must be provided with and use disposable gloves Masks must be worn by all customers: staff must wear a mask in all interactions with customers. Children under 6 years of age and subjects with health conditions that preclude the wearing of a mask are exempted from this rule Common areas must be cleaned and disinfected daily: cleaning and disinfection must be carried out twice daily according to the opening hours of the business Indoor areas should be aired: if technically possible, systems that recirculate air such as climate control system or air conditioners must be switched off Points of sale may be fitted with physical barriers, e.g., screen In all activities and phases, an interpersonal distance of at least 1 m must be maintained

**Table 2:** Use of personal protection equipment or adoption of specific safety measures by pharmacists who tested either negative or positive to the serological test.

Variable	Response	Pharmacists with Positive Serological Test n (%)	Pharmacists with Negative Serological Test n (%)
Mask (any type)	Yes	10 (4%)	274 (96%)
	No	0 (0%)	2 (100%)
		*p*-value = 1.00	
Face shield and/or safety glasses	Yes	0 (0%)	75 (100%)
	No	10 (5%)	201 (95%)
		*p*-value = 0.07	
Plexiglass protection barrier	Yes	9 (4%)	249 (96%)
	No	1 (4%)	27 (96%)
		*p*-value = 1.00	
Disposable gloves	Yes	9 (3%)	253 (97%)
	No	1 (4%)	23 (96%)
		*p*-value = 0.59	
Hand sanitizer	Yes	7 (3%)	257 (97%)
	No	3 (14%)	19 (86%)
		*p*-value = 0.03	
Decontamination of premises	Yes	4 (3%)	112 (97%)
	No	6 (4%)	164 (96%)
		*p*-value = 1.00	
Regular surface decontamination	Yes	8 (3%)	252 (97%)
	No	2 (8%)	24 (92%)
		*p*-value = 0.23	
Quota restrictions on admissions for customers	Yes	8 (3%)	260 (97%)
	No	2 (11%)	16 (89%)
		*p*-value = 0.13	

**Table 3:** Use of personal protection equipment or adoption of specific safety measures by pharmacists with a positive serological test.

Subjects	Cloth Mask	Surgical Mask	Filtered Mask FFP2 or FFP3	Face Shield	Safety Glasses	Plexiglass Barrier	Disposable Gloves	Disposable Gown	Hand Sanitizer (Gel or Liquid)	Regular Surface Decontamination	Decontamination of Premises by Certified Cleaners	Quota Restrictions on Admissions for Customers
1	no	yes	yes	no	no	yes	no	no	no	yes	no	yes
2	yes	yes	no	no	no	yes	yes	no	yes	yes	no	no
3	no	yes	no	no	no	yes	yes	no	no	yes	yes	yes
4	no	no	yes	no	no	yes	yes	no	no	yes	yes	yes
5	no	yes	no	no	no	yes	yes	yes	yes	yes	yes	yes
6	no	yes	yes	no	no	yes	yes	no	yes	yes	no	yes
7	no	yes	no	no	no	no	yes	no	yes	no	no	no
8	yes	no	no	no	no	yes	yes	no	yes	yes	no	yes
9	no	yes	yes	no	no	yes	yes	no	yes	yes	no	yes
10	no	yes	no	no	no	yes	yes	no	yes	no	yes	yes

## Data Availability

Data is contained within the article.

## Submission Declaration

This manuscript is an original paper, has not been published before, and is not under consideration for publication in another journal at the present time.
